# Beamforming Applied to Ultrasound Analysis in Detection of Bearing Defects

**DOI:** 10.3390/s21206803

**Published:** 2021-10-13

**Authors:** Thomas Verellen, Florian Verbelen, Kurt Stockman, Jan Steckel

**Affiliations:** 1 FTI-CoSys Lab, University of Antwerp, 2020 Antwerp, Belgium; thomas.verellen@uantwerpen.be; 2 Flanders Make Strategic Research Centre, 3920 Lommel, Belgium; 3 Department of Electrical Energy, Metals, Mechanical Constructions and Systems, Ghent University, 9000 Ghent, Belgium; florian.verbelen@ugent.be (F.V.); kurt.stockman@ugent.be (K.S.)

**Keywords:** acoustic signal processing, array signal processing, beamforming, microphone arrays, predictive maintenance

## Abstract

The bearings of rotating machinery often fail, leading to unforeseen downtime of large machines in industrial plants. Therefore, condition monitoring can be a powerful tool to aid in the quick identification of these faults and make it possible to plan maintenance before the fault becomes too drastic, reducing downtime and cost. Predictive maintenance is often based on information gathered from accelerometers. However, these sensors are contact-based, making them less attractive for use in an industrial plant and more prone to breakage. In this paper, condition monitoring based on ultrasound is researched, where non-invasive sensors are used to record the noise originating from different defects of the Machinery Fault Simulator. The acoustic data are recorded using a sparse microphone array in a lab environment. The same array was used to record real spatial noise in a fully operational plant which was later added to the acoustic data containing the different defects with a variety of Signal To Noise ratios. In this paper, we compare the classification results of the noisy acoustic data of only one microphone to the beamformed acoustic data. We do this to investigate how beamforming could improve the classification process in an ultrasound condition-monitoring application in a real industrial plant.

## 1. Introduction

Machine condition monitoring is a useful tool for ensuring safe and correct operation of equipment in large industrial plants. Condition monitoring enables predictive maintenance, which can reduce downtime of equipment and overall maintenance cost. Furthermore, when applied to milling operations, e.g., the state of the mill, it could extend the lifespan of the inserts, reduce cost and make way for a new industry standard of quality and health control [1].

Predictive maintenance has many definitions; often, it is referred to as monitoring the vibration of rotating machinery in an attempt to detect incipient problems and to prevent catastrophic failure [2]. When the overall condition of the machine declines, a change in its vibrations will become apparent, making it possible to use features such as the Root Mean Square (RMS) or peak values of the vibration signal to detect the onset of faults and plan maintenance [3,4].

In the field of condition monitoring, the focus often lies on bearing failures, which are usually the cause of a major failure resulting in a halt of production or major incidents on the work floor. A simple physical model of the bearing faults can be used to calculate the spectral signatures of these faults. These signatures can then be monitored closely to estimate the current state of the bearing.

However, accelerometers are contact sensors and need to be attached to the device under investigation. When the machine is not equipped with these sensors, the least intrusive way is to use magnets or wax to attach them. The downside is that in a lot of industrial environments, this is not a valid option for long-term monitoring since these connections are only possible on flat surfaces and should be checked regularly. Furthermore, the frequencies that can be investigated with magnet or wax-mounted accelerometers are limited [3,5]. Different mounting methods can change the signal transmissibility and damping rates of the captured signals which can lead to undesirable measured structural responses [5].

For this reason, we will investigate the use of ultrasonic sensors to monitor the condition of rotating machines and compare this to a traditional accelerometer. The advantage of using acoustic-based sensors is that these are non-contact sensors which can easily be placed in industrial plants without being intrusive at all. Since they are not placed on the machine itself, they are also less prone to breakage and therefore more interesting to use when looking into long-term non-intrusive condition monitoring.

In this paper, we will investigate bearing defects using the SpectraQuest Machinery Fault Simulator-Lite (MFS-LT) [6,7]. The MFS-LT makes it possible to simulate common machine faults such as unbalance, misalignment, resonance, bearing defects, crack shafts, fan rub and mechanical rub. When used to investigate bearing faults, it is most often used in combination with accelerometers which can be screwed into place.

In this paper, an array of microphones will be used as a sensor input into the classification pipeline and be compared to the classification results of a single accelerometer using the same pipeline. In particular, we will use the array of microphones to look into the audible and ultrasound spectrum ranging from around 0 Hz to 100 kHz. In traditional approaches to acoustic condition monitoring, most of the research effort has gone towards analyzing and utilizing the audible frequency range of the acoustic spectrum (from 0 Hz to 20 kHz) [8,9,10]. Acoustic signals can also be used to detect a more gradual deterioration of bearings, as described by Yani et al. [8] who used bearings that were soaked in H_2_SO_4_ for a varying number of days. Lu et al. [9] further expanded bearing analysis based on acoustics to account for the fact that these bearings are not always operated at constant speeds, further improving the use-case of sound signal analysis in an industrial environment. These audible frequencies are also investigated to classify the state of a face milling tool [10] proving the need to investigate these types of classification processes and if they would still function when executed with real-life data.

However, since a live industrial plant is often cluttered with noise, we were interested in the ultrasound spectrum as well. Shorter wavelengths make it possible to efficiently use beamforming techniques to reduce the vast amount of noise that is present when not working in a lab-type environment. Most research using audible frequencies also use some sort of deep learning [11] or Support Vector Machines (SVMs) [12,13] in the decision-making process, whereas others combine the recorded audio data with data captured from another sensor such as a current sensor [14]. However, these current sensors are also intrusive and not always available in an operational industrial plant. Therefore, we will focus only on the data recorded with our microphone array that can be placed in a minimally intrusive way, without the need of modifications to the machine.

Another solution to the noise-cluttered audible spectrum is to focus on acoustic emission of the machine under investigation [15]. Acoustic emission focuses on extremely high acoustic frequencies (typically 100 kHz to 1 MHz) that can only be measured with direct contact sensors, which again makes them not of interest for our application since the focus lies on contactless predictive maintenance. An entire overview between acoustic emission and vibration signals is available in [16] or a combination of acoustic emission and vibration data in [17].

Ultrasound has already been proven to detect faults quicker and more accurately compared to vibration data [18], especially when operating at low speeds [19]. When looking into ultrasound methods of condition monitoring, often expensive probes or heterodyne detection schemes are used to reduce the frequency [20,21,22], which are interesting in applications where a user would listen to the degraded ultrasound signal but not when the signal will be processed by an SVM where the loss of information during the heterodyne process could deteriorate the outcome. The focus of this paper will be on an application without the need for human intervention.

Early research proved the viability of airborne ultrasound to detect faults quicker and more accurately [19]. However, in the past few years, the focus changed from non-contact to contact ultrasound sensors or acoustic emission [23,24], which are more intrusive or expensive and not viable in an industrial environment. Therefore, this research will focus on non-contact ultrasound detection of bearing faults cluttered with real industrial noise and compared with more traditional vibration data. Furthermore, we will investigate the use of beamforming as an initial step to improve the results when using ultrasound to classify the different bearing faults.

As input for our SVM, we will use the spectral kurtosis [25], which is highly capable of detecting bearing faults and often used with vibration or current signals [26]. In this paper, only the spectral kurtosis is used as input for the classification process in an increasingly noisy environment. The classification results of the spectral kurtosis of vibration data are compared to the classification results of an ultrasound signal contaminated with real-life spatial noise recorded in an operational industrial plant. To improve the classification results of the acoustic data, a microphone array is used to perform a beamforming operation on the recorded signals, focusing the sensor in the direction of the faulty bearing. This improves the quality of the data used for condition monitoring by removing interference from surrounding sources.

The remainder of this paper is structured as follows: first, the data acquisition scheme and data processing steps are explained in Section 2.1 and Section 2.2, respectively. Next, the results are shown and discussed in Section 3. Next, in Section 4, the conclusion of the paper is stated. Finally, in Section 5, future work is briefly discussed.

## 2. Methodology

### 2.1. Data Acquisition

**Figure 1:**
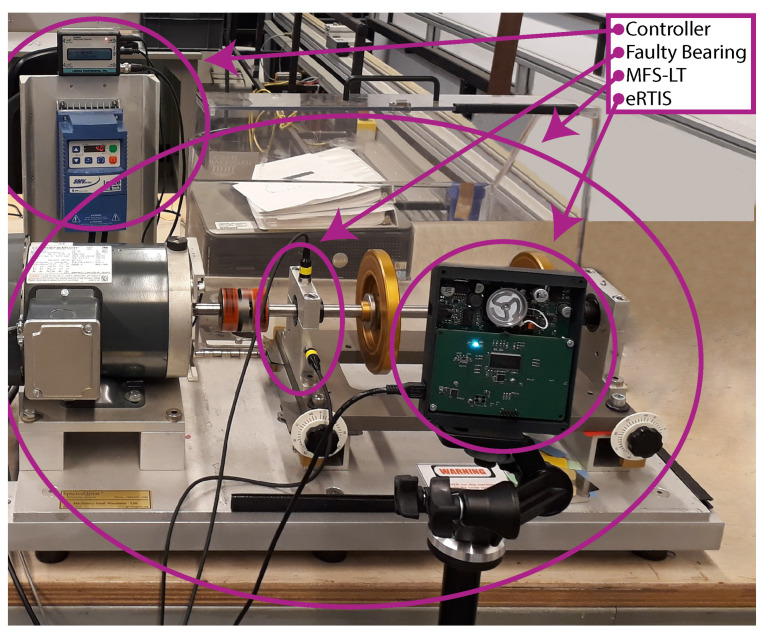
Experimental setup used to investigate the influence of beamforming to acoustic condition monitoring. The setup consists off an eRTIS pointed towards the faulty bearing of the Machinery Fault Simulator Lite. The controller is used to rotate the rotor shaft at a desired frequency.

The experiments were conducted using the Machinery Fault Simulator-Lite (SpectraQuest Inc., Richmond, VI, USA), during which the focus lies on bearing defects. In particular, we used faulty bearings containing a ball defect, inner ring defect, outer ring defect, and a combination of all the previously mentioned from the SpectraQuest Inc. 5/8${}^{\prime \prime }$ Bearing Fault Kit. Combined with the reference bearing, this results in five different classes for the classification process. The MFS-LT was used to rotate a shaft supported by two bearings at 20 Hz, while vibration signals were captured with an accelerometer at 51.2 kHz and acoustic data were recorded at 450 kHz. The acoustic data were captured using the embedded Real-Time Imaging Sonar (eRTIS) [27] developed by CoSys-Lab. This data were recorded in a quiet lab environment with a minimum of background noise to ensure clean data. The entire setup is shown in Figure 1.

**Figure 2:**
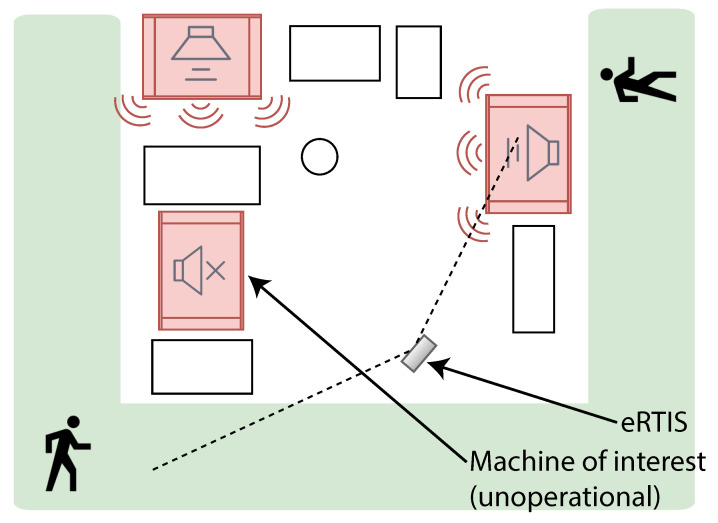
Experimental setup used to record the spatial background noise in an industrial plant. The red rectangles indicate the machines, the white rectangles indicate different types of furniture such as desks or storage racks. The green surrounding the setup indicates the path for people walking or operating industrial trucks such as forklifts.

To investigate the use of beamforming for condition monitoring, the same eRTIS was used to record background noise at an industrial plant that was in full operation. The eRTIS was placed in front of a machine that could be the subject of investigation and recorded background noise during the off-time of that machine. This was done to ensure realistic spatial background noise was captured where they did not take our presence into account. Figure 2 shows a simple ground plan of the location of the eRTIS. It is located in front of the machine of interest, surrounded by other machines which were generating interfering noise. The vibration data will also be contaminated with noise, which is additive white gaussian noise.

The great advantage of using an acoustic sensor such as the eRTIS is the fact that the installation can be outside of the enclosure of the machine, reducing the need to be resistant to harsh conditions such as water, oil or heavy vibrations. However, we designed a new enclosure that is resistant to splash water and dust, so it is robust enough for installation on an industrial site.

### 2.2. Data Processing

The recordings of the MFS-LT taken with the 32 synchronized microphone array of the eRTIS and an accelerometer, which all lasted around 3.7 s, were split up in 74 overlapping recordings lasting around 1 s. Each of the overlapping recordings

(1) $$X=\left[\begin{array}{cccc} {\vec{x}}_{1} & {\vec{x}}_{2} & \cdots & {\vec{x}}_{32} \end{array}\right]$$

of the eRTIS consists of 32 signals $\vec{x}$ for our 32 microphones. Each of the 32 signals are of length *M* lasting around 1 s, resulting in X being of size M×32. These overlapping recordings of the eRTIS were added with spatial noise $\vec{n}$ recorded in an industrial plant with varying Signal to Noise Ratios (SNRs). For the vibration data, white gaussian noise was added with varying SNRs. The noisy recordings of the eRTIS resulted in *Y*:

(2) $$\begin{array}{cc} Y & =X+k\cdot N,\\ Y & =\left[\begin{array}{cccc} {\vec{y}}_{1} & {\vec{y}}_{2} & \cdots & {\vec{y}}_{32} \end{array}\right],\\ N & =\left[\begin{array}{cccc} {\vec{n}}_{1} & {\vec{n}}_{2} & \cdots & {\vec{n}}_{32} \end{array}\right],\\ k & =\frac{∥{\vec{n}}_{1}∥}{∥{\vec{x}}_{1}∥}\times 10^{\left(0.05\times SNR\right)}, \end{array}$$

where *k* is a scaling factor dependent on the SNR; $∥{\vec{x}}_{1}∥$ and $∥{\vec{n}}_{1}∥$ denote the L2 norm of the signal and noise recording, respectively, where the first microphone was used as reference; and $\vec{n}$ has been trimmed to be of the same length *M* as the overlapping recordings. This was done for an SNR ranging from —20 to 20 dB to investigate the influence of beamforming to the classification process of the different recordings and investigate the possibility of using this as an initial step to improve current methods of acoustic condition monitoring.

To achieve this, two signals were formed from these acoustic recordings, namely a raw signal ${\vec{y}}_{R}$ and a beamformed signal ${\vec{y}}_{BF}$. For the raw signal ${\vec{y}}_{R}$, the recordings of only a single microphone (microphone 5) of the eRTIS were used with the added noise as in

(3) $${\vec{y}}_{R}\left[k\right]={\vec{y}}_{5}\left[k\right].$$

The beamformed signal ${\vec{y}}_{BF}$ consisted of applying Delay-And-Sum (DAS) [28] to the noisy microphone data of each recording. This beamformer will utilize the delays in arrival time between the different microphones to focus on the audio signal originating from our bearings and attenuate noise coming from elsewhere. This results in

(4) $${\vec{y}}_{BF}\left[k\right]=\sum_{n=1}^{32}{\vec{y}}_{n}\left[k+\tau_{n}\left(\phi \right)\right],$$

where $\tau_{n}\left(\phi \right)$ are the time-delays added to each channel to compensate for the angle-dependent difference in time-of-arrival caused by the geometry of the array and *k* ranging from 1 to *M*. Since the eRTIS was pointed directly at the faulty bearings, these time-delays are equal to zero.

**Figure 3:**
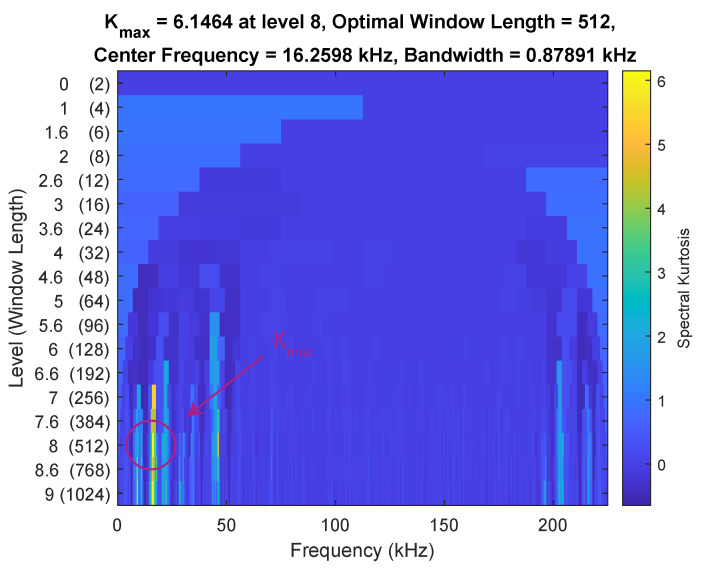
Nine-level kurtogram of noiseless beamformed acoustic data, captured at 450 kHz. The kurtogram was used to find the optimal window length for the spectral kurtosis based on the maximum value, indicated by the red circle.

Then, we calculate the short-time Fourier transform of the three acquired signals (raw acoustic data, beamformed acoustic data and accelerometer data):

(5) $$S\left[k,f\right]=\sum_{k=1}^{M}s\left[k\right]w\left[k-\tau \right]e^{-2\pi fk}$$

with s[k] being equal to either ${\vec{y}}_{R}\left[k\right]$, ${\vec{y}}_{BF}\left[k\right]$ or the accelerometer data ${\vec{y}}_{ACC}\left[k\right]$. w[t−τ] is the Hann function with a window size resulting in the highest spectral kurtosis value for the noiseless signal calculated with a kurtogram. This resulted in a window size *W* of 8, 256 and 512 for the accelerometer, raw data and beamformed data (Figure 3), respectively.

These STFTs were used to calculate the spectral kurtosis of these signals. Antoni [25] described the spectral kurtosis as a statistical tool which can indicate the presence of series of transients and their locations in the frequency domain and defined it as a normalized fourth-order spectral cumulant:

(6) $$SK\left[f\right]=\frac{\langle |S\left[k,f\right]{|}^{4}\rangle }{\langle |S\left[k,f\right]{|}^{2}\rangle }-2,$$

where SK[f] is the spectral kurtosis around a certain frequency *f*, and S[k,f] the time-frequency envelope acquired by decomposing the input signal using the Wold-Cramer representation. This envelope is averaged in time, denoted by 〈⋅〉. For each of the 74 overlapping segments, the corresponding spectral kurtosises were calculated. The number of frequency points for these spectral kurtosises is equal to 1+4W. For the accelerometer data with a window of 8 samples and an overlap of 80%, this results in a matrix of size [74×(1+4×8)]=[74×33]. For the raw acoustic data and beamformed acoustic data, the size is equal to [74×1025] and [74×2049], respectively.

Since four recordings are available for each of the five classes that are split up in 74 overlapping segments, the size of the obtained datastream is [4×5×74×33]=[1480×33] for the vibration data, [1480×1025] for the raw acoustic data and [1480×2049] for the beamformed acoustic data. The resulting data going into the machine learning pipeline are obtained by a concatenation of the spectral kurtosises:

(7) $$D={\left[\begin{array}{cccc} SK_{1}\left[f\right] & SK_{2}\left[f\right] & \cdots & SK_{1480}\left[f\right] \end{array}\right]}^{T}$$

in which the length of the spectral kurtosis varies between 33 for the vibration data, 1025 for the raw acoustic data and 2049 for the beamformed data. The datastreams get split up, where 740 out of 1480 rows from *D* will be used to train the SVM ($D_{tr}$), and the remaining 740 will be used to test the classification ($D_{te}$). The entire process will be 20-fold cross validated.

Next, a Principal Component Analysis (PCA) [29] basis is calculated from the training data of all five classes $D_{tr}$ to perform dimensionality reduction [30]. PCA will transform the data to a new coordinate system that orders its variance from high to low on its coordinates, enabling us to only select the first components and reducing the dimension of the data with an optimized variance. t-Distributed Stochastic Neighbor Embedding was used to experimentally determine the optimal size of the projection basis for the PCA. This resulted in using the 20 most prominent eigenvectors to construct a projection basis $V_{PCA}$ of size [33×20] for the accelerometer data, [1025×20] for the raw acoustic data and [2049×20] for the beamformed data. The dimensionality-reduced feature vectors can then be found by the matrix-product between the data-matrices and the PCA-matrix as in

(8) $$D_{tr}^{V}=D_{tr}\cdot V_{PCA},\;D_{te}^{V}=D_{te}\cdot V_{PCA},$$

where $D_{te}$ is the test data of all five classes which is of the same size as $D_{tr}$. These dimensionality-reduced feature vectors are then classified using a multi-class SVM [30] with polynomial kernels of order five and automatic kernel scaling. The SVM will construct a set of hyperplanes which can then be used for classification. These hyperplanes are trained such that the distance between them and the nearest training point is as high as possible. This entire process was repeated 41 times for an SNR ranging from —20 to 20 dB.

## 3. Results and Discussion

**Figure 4:**
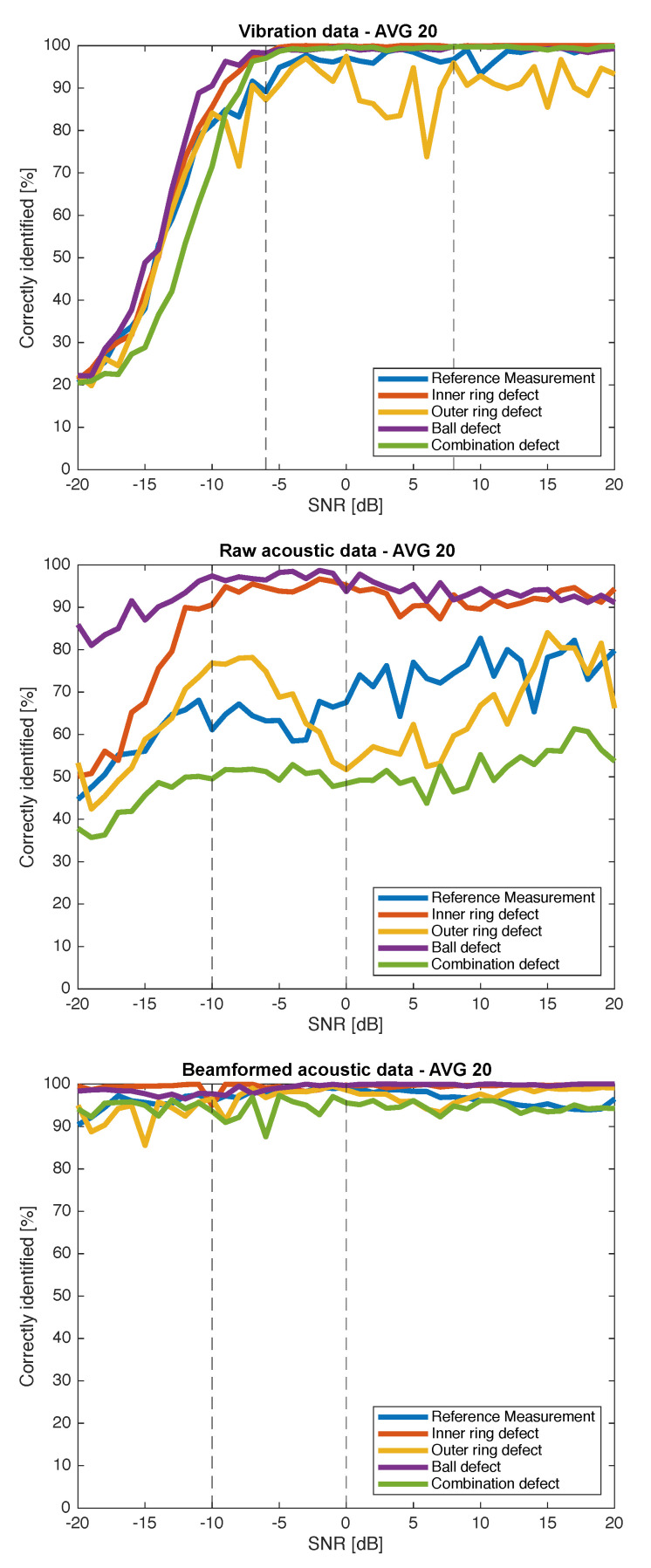
Percentage of correctly identified samples in function of the SNR. The data in the top graph are data captured with an accelerometer. The data in the middle graph are raw data taken from one microphone of the array of the eRTIS. To classify the different defects in the bottom graph, beamformed data were used. The data consist of a reference measurement of a healthy bearing as well as a bearing with an inner ring defect, outer ring defect, ball defect and a combination of defects. The dashed lines indicate realistic SNRs (realistic worst and average case) for the vibration and acoustic data. The training and classification was repeated 20 times and averaged.

Figure 4 shows an overview of the percentage of correct predictions for the different defects and reference measurement. To gain insight into the data shown in Figure 4, we marked realistic SNRs that could appear in an industrial environment with two dashed lines, one for a realistic worst and average case. Although it is hard to measure the SNR of bearing defects in an industrial setting, recent literature focused on the detection of bearing defects in trains with acoustic data and often used values between —10 and 0 dB [31,32], while realistic SNRs of vibration data lie between —6 and 8 dB [33].

**Figure 5:**
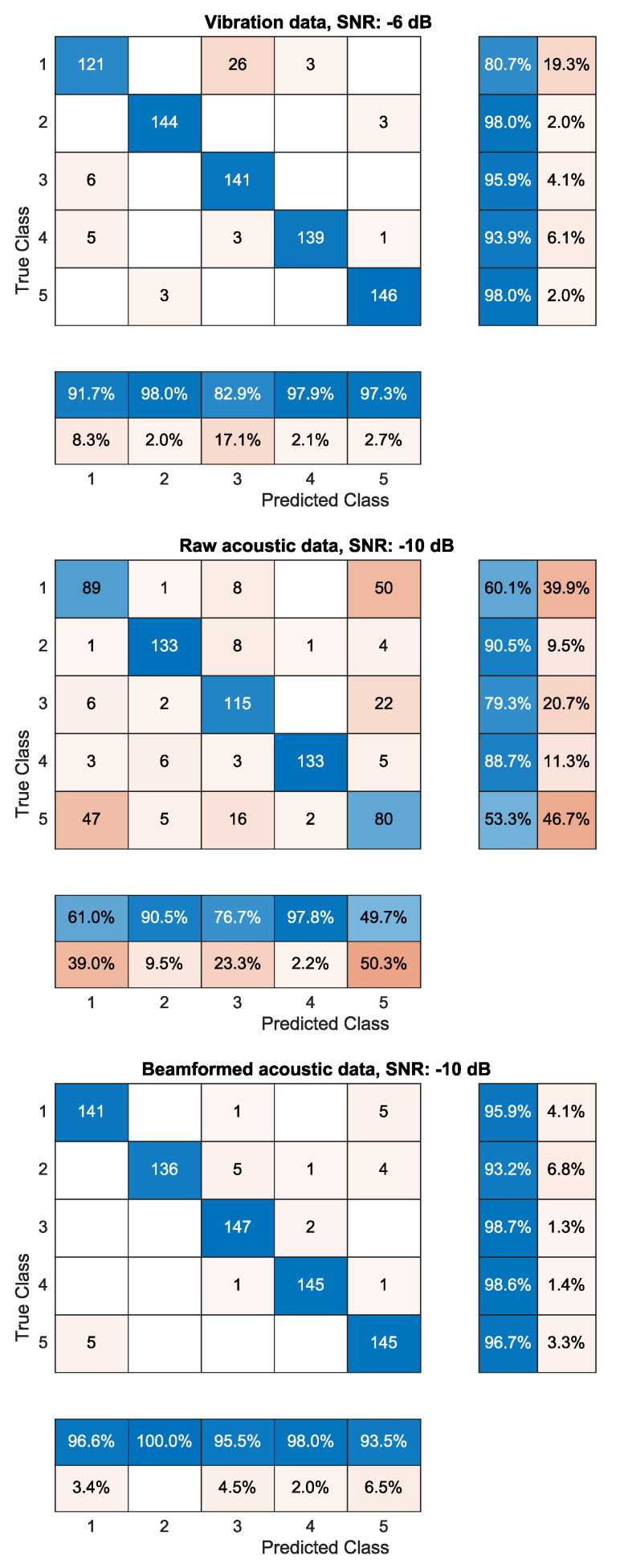
Confusion matrices of the vibration data (top matrix), raw acoustic data (middle matrix) and beamformed acoustic data (bottom matrix) for realistic worst SNRs (—6 dB for vibration data, —10 dB for acoustic data). The data consist of a reference measurement of a healthy bearing (class 1) as well as a bearing with an inner ring defect (class 2), outer ring defect (class 3), ball defect (class 4) and a combination of defects (class 5). Classification is achieved with an SVM that uses a spectral kurtosis reduced to 20 dimensions as input.

**Figure 6:**
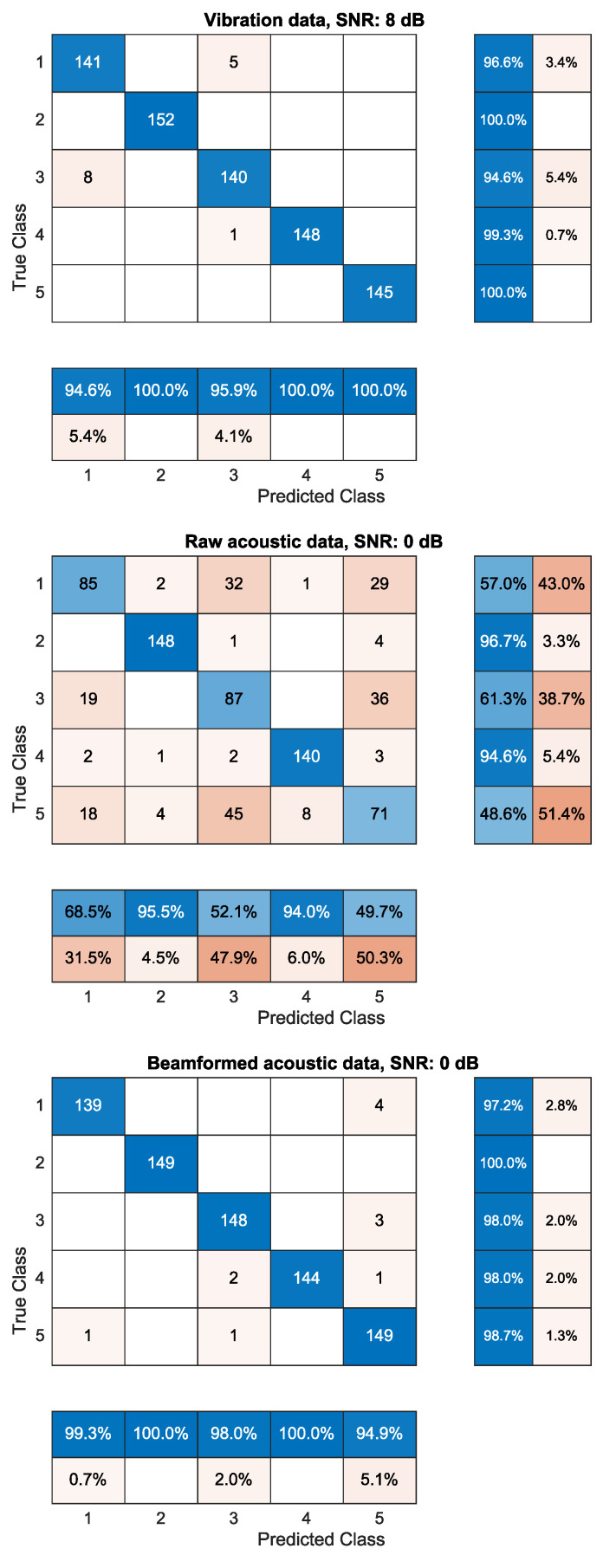
Confusion matrices of the vibration data (top matrix), raw acoustic data (middle matrix) and beamformed acoustic data (bottom matrix) for realistic average SNRs (8 dB for vibration data, 0 dB for acoustic data). The data consist of a reference measurement of a healthy bearing (class 1) and a bearing with an inner ring defect (class 2), outer ring defect (class 3), ball defect (class 4) and a combination of defects (class 5). Classification is achieved with an SVM that uses a spectral kurtosis reduced to 20 dimensions as input.

Figure 4 shows an overview of the percentage of correct predictions for the different defects and reference measurement. To gain insight into the data shown in Figure 4, two SNRs (a realistic worst and average case) were chosen to display the confusion matrices. These results are available in Figure 5 and Figure 6, respectively.

Figure 4 makes it clear that beamforming has a positive influence on the classification of our data. The correctly identified recordings are higher for every available defect and every tested SNR. The raw data further show that their classification results are not suitable for real-life applications. The prediction rate of the healthy bearing for the raw data does not exceed 80%, even with extremely high SNRs. The beamformed data, on the other hand, correctly predict the healthy bearing at least 90% of the time, even when the SNR is equal to —20 dB.

The latter results are comparable with vibration data which also achieve high prediction results even in very noisy environments up to —6 dB. For SNRs lower than —6 dB, the prediction results of the vibration data start to plummet. However, in most realistic applications, the SNR is above —6 dB. These results could be further improved by using a second accelerometer as proven in [34], where two accelerometers are used to classify different bearing faults along with unbalance defects. Another way of improving this would be to use Extreme Learning Machine algorithms as used in [35], where classification rates of up to 100% are achieved in the absence of added noise. However, we use a more transparent SVM to focus on the improvement beamformed acoustic data can provide compared with vibration data.

To gain more insight into the classification data, the confusion matrices are shown in Figure 5 and Figure 6 for an SNR in a realistic worst-case scenario (—6 dB for the accelerometer and —10 dB for the acoustic data) and a realistic average case scenario (8 dB for the accelerometer and 0 dB for the acoustic data), respectively.

The top matrix in Figure 5 shows the confusion matrix for the vibration data for an SNR of —6 dB. The middle and bottom matrices display the confusion matrices for the raw and beamformed acoustic data for an SNR of —10 dB, respectively. Comparing the raw and beamformed acoustic data, it can be seen that the beamformed signal is more capable in correctly identifying the different defects. More importantly, the beamformed signal in a worst-case scenario is capable of correctly predicting the healthy bearing in 96.6% of the cases compared to 61.0% for the raw data. The classification results of the beamformed signal are comparable to that of the more traditional vibration data, for which the prediction rate is slightly lower at 91.7%.

Overall, classes 2 and 4 (inner ring defect and ball defect, respectively) are predicted most accurately with 90.5% and 97.8% for the raw acoustic data in a very noisy environment. This is because the fault frequencies of these two defects are the highest and lowest of all tested defects and the fault frequency of class 3 (outer ring defect) lies in between the previous two, making it easier to wrongly classify the recording of this defect.

More importantly, the beamformed data are able to outperform the vibration data when using only the spectral kurtosis as feature. The same can be seen in Figure 6, where the beamformed data are able to correctly predict the reference measurement in 99.3% compared to 94.6% of the cases when using a better, but still realistic, SNR. However, it should be stated that this is in this particular case, and Figure 4 clearly shows that the vibration data for higher SNRs are able to outperform the beamformed data.

Research exists using accelerometers and spectral kurtosis features achieving better classification results using more advance machine learning algorithms [35] or under masking noise originating from a faulty gearbox [36]. However, this paper is focused on using and comparing the classification results when using the spectral kurtosis on vibration data as well as acoustic signals, proving that using the signal of only one microphone cannot compete with that of an accelerometer. However, by preprocessing the acoustic data by combining multiple microphones to beamform and focus on the audio originating from the faulty bearing, ultrasound can achieve similar results as vibration data.

## 4. Conclusions

The MFS-LT was used to gather vibration and acoustic data of different bearing defects. These data were added with real spatial noise recorded in a live industrial plant in the case of the acoustic data and white gaussian noise for the vibration data. The data were classified using a spectral kurtosis reduced to 20 dimensions and an SVM. It was shown that the beamformed data were able to more correctly identify the reference measurement and defects even in extremely low SNR scenarios. Over 90% of the predicted reference measurements were correct compared to only 60% when the data were not beamformed and with worse performance for the different defects.

More importantly, for these realistic SNRs, the beamformed data were able to perform just as well as an accelerometer with a classification rate of at least 96.6%, while being a more universal sensor which is easier to apply in realistic industrial environments.

While current research often focuses on using vibration measurements or ultrasound gathered from contact sensors or acoustic emission, our research suggests using non-contact ultrasound sensors can achieve similar results when preprocessed with a beamforming step. Where these contact sensors can be intrusive or prone to breakage, the benefit of using these microphone arrays is that they can be installed outside of the enclosure at a safe distance in a lesser harsh environment. Even when cluttered with spatial noise recorded at an industrial plant, the beamformed data are able to clearly distinguish the different bearing defects when using the spectral kurtosis.

## 5. Future Work

Research often focuses on experiments performed in lab environments, while this study used data of bearing defects captured in a lab environment and cluttered them with noise from a live industrial plant. This method is ideal to better understand the captured data and how they react in an operational company. To continue this research, we should gather bearing data originating from an industrial machine instead of the MFS-LT, where the acoustic signal produced by the faulty bearings is attenuated by the enclosure or masked by other parts of the machines such as in [36]. Since the spectral kurtosis level is dynamically chosen, it could also be of interest to record data from different types of machines and investigate a more universal use of this method. Following this, we could apply more advanced techniques as described by Tian et al. [36] or Udmale et al. [35] who further improved the usability and accuracy of bearing classification with the spectral kurtosis of vibration data.

## Abbreviations

The following abbreviations are used in this manuscript:

MFS-LT	Machinery Fault Simulator-Lite
SVM	Support Vector Machine
eRTIS	embedded Real-Time Imaging Sonar
DAS	Delay-And-Sum
STFT	Short-Time Fourier Transform
PCA	Principal Component Analysis
